# Systematic content analysis of patient evaluations of START NOW psychotherapy reveals practical strategies for improving the treatment of opioid use disorder

**DOI:** 10.1186/s12888-020-03024-x

**Published:** 2021-01-10

**Authors:** Albert Yi-Que Truong, Brian Fabian Saway, Malek H. Bouzaher, Mustafa Nawroz Rasheed, Sanaz Monjazeb, Soleille Dorothy Everest, Susan Linda Giampalmo, David Hartman, Cheryl Hartman, Anita S. Kablinger, Robert L. Trestman

**Affiliations:** 1 Virginia Tech Carilion School of Medicine, 2 Riverside Circle, Roanoke, VA 24016 USA; 2 Carilion Clinic Psychiatry & Behavioral Medicine, 2017 S. Jefferson Street, 1st Floor Administrative Suite, Roanoke, VA 24014 USA

**Keywords:** Opioid use disorder, Medication-assisted treatment, Office-based opioid treatment, Implementation science

## Abstract

**Background:**

Clinical trials provide consistent evidence for buprenorphine’s efficacy in treating opioid use disorder (OUD). While the Drug Addiction Treatment Act of 2000 requires physicians to combine medication-assisted treatment (MAT) with behavioral intervention, there is no clear evidence for what form or elements of psychotherapy are most effective when coupled with MAT to treat OUD. This investigation involves focus groups designed to collect patient opinions about a specific psychotherapy, called START NOW, as well as general beliefs about various elements of psychotherapy for treating OUD. Our analysis reveals trends about patient preferences and strategies for improving OUD treatment.

**Methods:**

Subjects included patients enrolled in buprenorphine/naloxone MAT at our institution’s office-based opioid treatment program. All subjects participated in a single START NOW group session, which was led by a provider (physician or nurse practitioner trained and standardized in delivering START NOW). Consented subjects participated in satisfaction surveys and audio-recorded focus groups assessing individual beliefs about various elements of psychotherapy for treating OUD.

**Results:**

Overall, 38 different focus groups, 92 participation events, and 44 unique subjects participated in 1-to-6 different START NOW session/audio-recorded focus group sessions led by a certified moderator. Demographic data from 36/44 subjects was collected. Seventy-five percent (33/44) completed the START NOW Assessment Protocol, which revealed self-reported behavioral trends. Analysis of all 92 START NOW Satisfaction Questionnaire results suggests that subjects’ opinions about START NOW improved with increased participation. Our analysis of audio-recorded focus groups is divided into three subsections: content strategies for new psychotherapies, implementation strategies, and other observations. For example, participants request psychotherapies to target impulsivity and to teach future planning and build positive relationships.

**Conclusions:**

The results of this study may guide implementation of psychotherapy and improve the treatment of OUD, especially as it relates to improving the modified START NOW program for treating OUD. Our study also reveals a favorable outlook of START NOW with increased participation, suggesting that any initial reticence to this program can be overcome to allow for effective implementation.

**Supplementary Information:**

The online version contains supplementary material available at 10.1186/s12888-020-03024-x.

## Background

In the United States, opioid use is pervasive with 10.1 million people (or 3.7% of the population) aged 12 or older in 2019 misusing opioids in the past year [1]. According to data from the 2019 National Survey on Drug Use and Health, access to addiction treatment is limited, and only 18.1% of people aged 12 or older with a past year opioid use disorder (OUD) received medication-assisted treatment (MAT) in the past year for opioid misuse [2]. This is according to the Substance Abuse and Mental Health Services Administration (SAMHSA), which estimates that about 1.6 million people 12 or older qualify for the diagnosis of OUD based on the *Diagnostic and Statistical Manual of Mental Disorders, Fifth Edition* [2].

The treatment of OUD is complicated by the fact that many patients have psychiatric comorbidities, psychosocial challenges such as a history of incarceration, and socioeconomic challenges such as unemployment and homelessness [3–7]. Moreover, opioid misuse is not only related to other substance use but also associated with increased rates of comorbid depression and anxiety disorders [8–10]. For example, one study found that 47.1% of individuals with prescription opioid dependence were also diagnosed with comorbid mood or anxiety disorders [10].

Prevalence data from SAMHSA’s 2019 report provides further evidence that substance use is more frequent in adults (> 18 years old) with diagnosed mental illness [2, 11, 12]. Specifically, in the United States, 13.8% with serious mental illness and 8.8% with any mental illness misused opioids in the past year compared to 2.5% in adults without mental illness [2]. Because of these impairing, widespread, and numerous comorbidities, many groups of clinicians have suggested that, at the very least, access to screening and treatment of these comorbidities in individuals with OUD is important for improving overall health and likely opioid treatment success [6, 11, 13]. From our experience and perspective, there is a need for integrated, comprehensive interventions—that take into account all co-existing comorbidities—in order to effectively treat OUD.

In our office-based opioid treatment setting, we propose that medication-assisted treatment should be paired with a more holistic psychotherapy [14]. Therefore, we suggest that such a comprehensive intervention is a modified version of START NOW targeted specifically towards the substance use patient population. START NOW is a skills-based psychotherapy that was originally implemented and studied in the Connecticut Correctional Health Research Program with support from a National Institute of Justice grant (NIJ 2002-IJ-CX-K009) [15].

START NOW is a free, manual-guided skills training psychotherapy that integrates cognitive behavior therapy, motivational interviewing, trauma-informed care, and elements of cognitive neuro-rehabilitation [15]. Entirely available in the public domain, START NOW was originally designed for low-resource settings and as a psychotherapy for incarcerated individuals who present with mood dysregulation, impulsivity, aggression, and interpersonal discord. A retrospective cohort analysis of 850 patients in state prison demonstrated a significantly reduced risk of disciplinary infractions and future psychiatric inpatient days with a dose response effect [16, 17]. Furthermore, START NOW has been associated with reduced risk of criminal recidivism in an evaluation of a specialized alternative-to-incarceration program for individuals with serious mental illness and co-occurring substance use disorder [18].

Our primary research question is: what are patients’ opinions about START NOW therapy and office-based opioid treatment (OBOT)? This article provides a detailed analysis on patient evaluations of START NOW psychotherapy. For this study, START NOW has been modified for the substance use disorder patient population and is currently being studied in a clinical trial for its effectiveness in treating OUD when paired with buprenorphine/naloxone medication-assisted treatment. At our institution (Carilion Clinic, Roanoke, VA) and with the resources available to us, the treatment of opioid use disorder is near-exclusively offered in the form of buprenorphine/naloxone MAT in the setting of outpatient OBOT. Group therapy, along with urine drug screens and buprenorphine/naloxone distribution, occurs once a week for a majority of patients. Therefore, this study seeks to explore how we can maximize the benefit of psychotherapy administered to our patients. Specifically, we plan on using this collected feedback to possibly modify START NOW and improve its delivery so that it is more effective and culturally appropriate for the OUD patient population. In our opinion, MAT OBOT programs may not always be appropriate treatment for every patient; for example, such limitations include but are not limited to variability in effectiveness of psychotherapy administered, patients who may benefit from inpatient treatment rather than outpatient services, and patients who may benefit from more frequent follow-up than weekly sessions [19, 20]. Nonetheless, MAT OBOT programs have been shown to be effective for treating OUD and are deserving of further study [21–24].

## Methods

### Setting

This research occurred at a single-center outpatient office-based opioid treatment program with the Department of Psychiatry and Behavioral Medicine at Carilion Clinic in Roanoke, Virginia.

### Study participants and inclusion/exclusion criteria

Participants selected for this study were patients of both genders and over the age of 18 who were enrolled in and undergoing buprenorphine/naloxone (Suboxone, Indivior Inc.) MAT for OUD at our institution. Patients excluded from this study include minors under the age of 18 and those with psychiatric or medical co-morbidities requiring inpatient hospitalization.

### Intervention

This investigation involves a pilot study of START NOW’s structured 32-session OBOT program (Table 1). The 32 sessions of START NOW are divided into 4 units. Sessions are meant to be administered once weekly for 32 weeks or two sessions per week for 16 weeks. Unlike the START NOW program originally applied in the forensic psychiatry and correctional setting, our version of START NOW psychotherapy has been significantly modified for treating substance use disorders [15]. For example, language about incarceration and references to inmates were removed, and anecdotes were modified to be more culturally appropriate and applicable to the SUD patient population.

**Table 1:** START NOW Psychotherapy

START NOW Psychotherapy		
**Units**	**Session #**	**Session Title**
**Unit 1 – My Foundation: Starting with Me** (10 sessions)	1	Understanding START NOW Therapy & Why It Starts with Me
	2	Focusing Skills
	3	Open & Balanced Attitude
	4	ABC Patterns
	5	Accepting Myself
	6	Accepting My Situation
	7	Self-Care Skills
	8	My Spiritual Self
	9	Identifying & Developing my Values
	10	Respecting my Personal Boundaries
**Unit 2 –My Emotions: Dealing with Upset Feelings** (8 sessions)	11	My Emotions & Feelings, part 1
	12	My Emotions & Feelings, part 2
	13	Coping with Upset Feelings through Actions
	14	Coping with Upset Feelings through Thoughts & Imagery
	15	Recognizing & Coping with Depression
	16	Coping with Anger
	17	Coping with Worry & Anxiety
	18	Coping with Loss & Grief
**Unit 3 – Me & Others: Building Positive Relationships** (8 sessions)	19	Beginning Positive Relationships
	20	Active Listening
	21	Assertiveness Skills
	22	Responding to Feedback
	23	Increasing my Support System
	24	Recognizing & Avoiding Negative Relationships
	25	Setting Boundaries
	26	Coping with Rejection
**Unit 4 –The Future Me: Continuing my Path to Success** (6 sessions)	27	Believing in my Future
	28	Setting & Making My Goals
	29	Problem Solving
	30	Setting & Reaching Educational Goals
	31	Setting & Reaching Vocational Goals
	32	Celebrating & Continuing My Progress

### Procedures

#### Overview

During August to October 2017, our research subjects participated in a single START NOW structured session, which was led by a trained provider (a medical doctor or nurse practitioner trained and standardized in delivering START NOW psychotherapy). Immediately after a psychotherapy session, subjects volunteered to consent and to participate in our study afterwards, which involved both paper surveys and an audio-recorded focus group session led by a trained, standardized moderator. Study participants who completed their surveys and a focus group received a small gift card reward for their participation.

#### Surveys

**Fig. 1:**
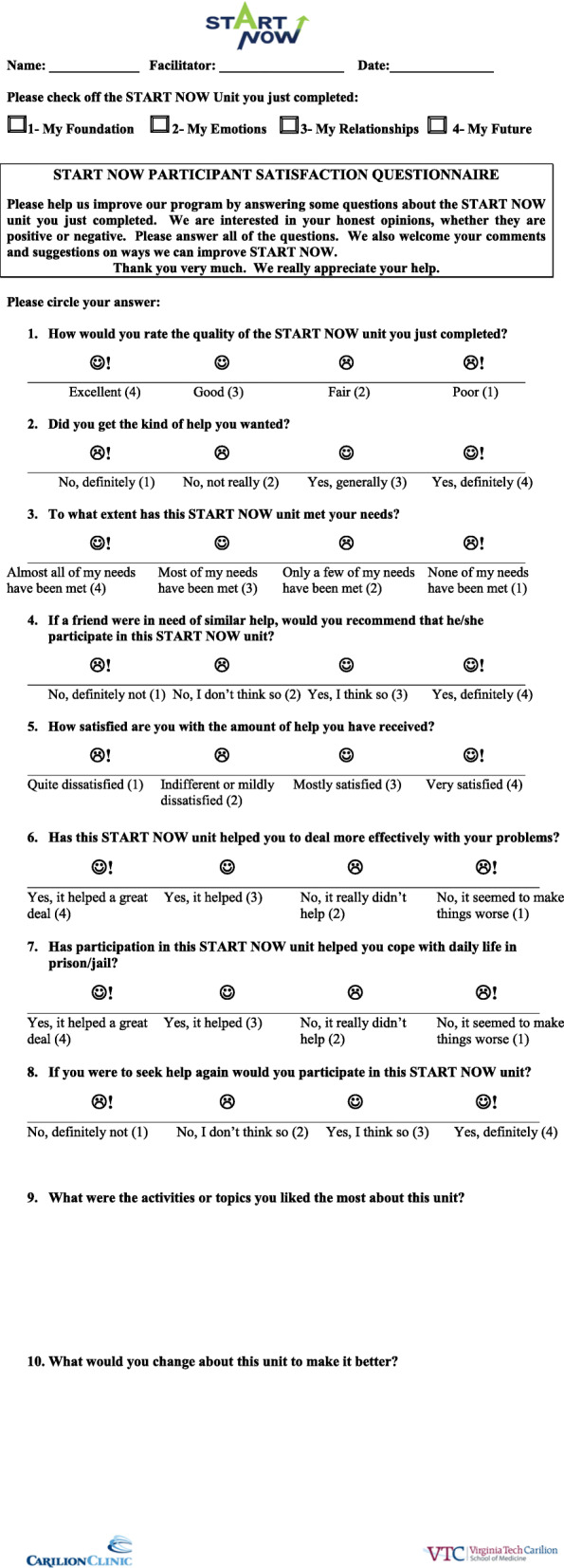
START NOW Questionnaire Survey. This is a sample image of the START NOW questionnaire

Every subject’s first participation event involves surveys consisting of: a demographic survey, START NOW Satisfaction Questionnaire, and the START NOW Assessment Protocol (SNAP). Any additional participation events only involve the START NOW Satisfaction Questionnaire as the demographic data and SNAP has already been collected. Subjects were able to participate in multiple START NOW sessions as long as each START NOW session was unique. Neither the START NOW Satisfaction Questionnaire nor the SNAP have been used in the clinical environment or evaluated in a research setting prior to this study (Figs. 1 & 3).

Despite the utility of assessments like the Barratt Impulsiveness Scale (BIS), Buss & Perry Aggression Questionnaire (BPAQ), and Inventory of Interpersonal Problems (IIP), the length of these assessments may be a barrier for both researchers and participants [25–27]. Therefore, the START NOW Assessment Protocol (SNAP), which remains to be validated in a clinical trial, was developed as an abbreviated tool. In this investigation, SNAP is being piloted for the first time in order to reveal general trends about our study population with regards to executive functioning, mood, impulsiveness, etc. This may provide baseline data for future investigations with this same population at our institution.

#### Focus groups

Moderated focus groups were designed to ask questions related to the five components of the START NOW psychotherapy program. These five components are: the real life practice exercises (equivalent to homework for participants), the in-session practice exercises (training exercises held during a psychotherapy session for developing focusing skills or practicing functional analysis of behavior), the specific lesson of the day, the participants’ view of the clinician, and the participants’ overall impression of START NOW. The primary purpose of the focus group questions is to collect participants’ opinions of the five components of delivering START NOW psychotherapy. See Table 2 for a full list of focus group questions, which were developed for this study. A description of the data analysis process for the survey data and focus group data is described below. Furthermore, a description of the standardization process for the START NOW clinicians, focus group moderators, and data analysis is described below.

**Table 2:** List of Focus Group Questions

Component of START NOW	Focus Group Questions
**View of Real Life Practice Exercises**	- Is it likely or unlikely for you to practice some of the take home exercises? - How can the facilitator encourage you to do the take home exercises? - Do you like or dislike having practice exercises to do at home?
**View of in- Session Practice Exercises**	- What content in the session could have been improved? - Was the worksheet during the START NOW session helpful or not helpful? Please explain.
**View of Specific Lesson of the day**	- Was there a concept that you found confusing or unclear? Please describe. - Was this lesson useful? - How could this lesson be better taught?
**View of Clinician**	- If the concept was confusing due to delivery, how could the delivery have been changed? - What could the facilitator have done to make you feel more involved and engaged? - What advice would you give the facilitator?
**Overall Impression of START NOW**	- How did your START NOW session compare to the other types of groups that you have been in before? - Do you think that you like START NOW more or less? - Sometimes, we’re not in the mood to participate. Was this true for you at any time during your START NOW session? - What is your overall impression of START NOW?

### Outcome measures

In addition to collecting participant demographics, the START NOW Satisfaction Questionnaire was used as a standardized approach to evaluate participants’ opinion about START NOW. This satisfaction questionnaire involves a 4-point numerical scale with 4 being “excellent” or “yes, definitely” and 1 being “poor” or “no, definitely.”

As mentioned previously, the results of the SNAP are used to reveal general trends about our study population with regards to executive functioning, mood, impulsiveness, etc. and may provide baseline data for future investigations with this same population at our institution. Outcome measures from SNAP are also numerical, involving a 5-point scale with 4 being “always” and 0 being “never.”

Traditionally, audio-recorded data is transcribed verbatim from audio-recording to an entirely text-based format [28–30]. However, in order to improve the efficiency of researchers and the analytical utility of the audio-recorded data, the recordings we collected from focus groups were transcribed to text-based format using a novel methodology. The first critical distinction with this methodology is as follows: researchers transcribing the audio recordings were instructed to copy every new idea (any opinion stated for the first time) verbatim. However, for every subsequent repetition of the same idea, researchers were instructed to make note of this repetition; in other words, copying the same idea twice is not necessary, but it should be noted that a participant mentioned an idea two or more times.

To facilitate in this process, a novel data transcription tool was created, which consists of various components of START NOW as described previously in the “Procedures: Focus Group” subsection. This represents the second critical distinction of our methodology as participants’ statements are then categorized into these specific components. Moreover, each component is further stratified into a five-point scale, which represents the strength of the opinion (strongly favorable, favorable, mixed, critical, or extremely critical). Each participant comment was rated based on this scale in order to capture the participants’ true opinion. In other words, researchers were trained and standardized in their approach to interpret the tone, voice, and statement of each participant in order to rate every comment into this five-point scale.

### Data analysis

For this study, we performed box plot analyses for the START NOW Satisfaction Questionnaire data. A systematic content analysis was performed on patient evaluations collected through audio-recorded focus groups to reveal participant opinions about START NOW specifically and OUD psychotherapy treatment in general. Data was exclusively analyzed on Microsoft Excel, which allowed us to generate all of the following: means of responses, percentages, interquartile ranges, and box plot diagrams.

For the demographic and SNAP data, percentages of responses were calculated. The START NOW Satisfaction Questionnaire involves 8 questions with responses on a four-point scale (i.e., 4 = excellent or yes definitely; 3 = good or yes I think so; 2 = fair or no I don’t think so; 1 poor or no definitely not). START NOW Satisfaction Questionnaire data was stratified based on the frequency of START NOW participation and then analyzed by calculating the range, mean, and interquartile range of responses.

As described previously in the “Outcome Measures” subsection, content analysis of audio-recorded data was performed by separating questions and answers based on the five START NOW components described above: the real life practice exercises, the in-session practice exercises, the specific lesson of the day, the participants’ view of the clinician, and the participants’ overall impression of START NOW. Researchers then stratified each participants’ comments based on a five-point scale, which represents the strength of the opinion (strongly favorable, favorable, mixed, critical, or extremely critical). Each participant comment was rated based on this scale in order to capture the participants’ true opinion. In other words, researchers were trained and standardized in their approach to interpret the tone, voice, and statement of each participant in order to rate every comment into this five-point scale. Of note, the “overall impression of START NOW” category is a rating entirely determined by the researcher responsible for transcribing the audio recording per a particular focus group. This is the researcher’s interpretation of a participant’s overall impression based on their feedback provided over the course of a focus group. For all these categories, the range, mean, and interquartile range of responses were computed.

### Standardization

Methodical measures were taken to standardize START NOW clinicians who piloted START NOW sessions, moderators who led the following focus groups, and researchers who transcribed the audio-recorded data for analysis.

In the end, each START NOW session piloted was overseen by one of seven different providers—either an attending medical doctor or a psychiatric nurse practitioner who is certified to prescribe buprenorphine/naloxone for MAT for OUD by the Virginia Department of Health. Some pilot sessions were co-run by resident physicians in psychiatry—to support resident training in psychotherapy efforts—but all groups were ultimately overseen by an attending physician. All clinicians who ran pilot sessions of START NOW for this study participated in and received certification after completing a two full-day comprehensive training course for START NOW psychotherapy. Prior to the training course, clinicians were expected to thoroughly understand all the START NOW materials, and thus, the training course mostly consisted of role-playing and practice exercises. This level of training and certification is consistent with the existing standard for training clinicians in START NOW for the correctional psychiatry setting.

Focus groups were facilitated by eight different medical students familiar with opioid use disorder research. Each focus group facilitator was trained to be culturally sensitive, objective, non-judgmental moderators based on the START NOW Facilitator Manual [15] and a Focus Group Discussion/Question Guide (Supplementary Fig. 1). In order to qualify to be a moderator, each focus group facilitator was required to: participate in two training sessions each two hours in length and sit in and shadow a focus group being led by one of the study lead investigators. These training sessions consisted of reviewing the first 26 pages of the START NOW Facilitator Manual, which describes START NOW and provides information about how to engage with patients, especially when dealing with challenging topics and facilitating participation [15]. All six researchers who helped transcribe the audio-recorded data received extensive training about how to listen and record data accurately and consistently between researchers. This training involved reading and understanding a standardized manual and passing a sample “test,” which required that all researchers listen to the same focus group audio recording and then transcribe this recording. A study investigator then reviewed each transcription to determine accuracy and consistency, which was achieved by all six researchers on first attempt.

## Results

### Overview

Almost all START NOW’s 32 unique sessions were piloted with following focus-groups except for sessions 15, 18, 26, and 31. These sessions were not evaluated through focus groups due to a lack of available, consenting participants. Some START NOW sessions were deemed to be especially important because they introduce key skills—focusing skills and the ABC (Activator, Behavior, Consequence) model for functional analysis of behavior—that are emphasized throughout the START NOW program. As a result, Session 1 and 2 (“Understanding START NOW Therapy & Why It Starts with Me” and “Focusing Skills,” respectively) and Session 4 (“ABC Patterns”) were piloted more frequently (7 times each). Note that Sessions 1 and 2 were combined into a single session for the purposes of this study. In the end, our study piloted 38 different focus groups with seven different clinicians.

### Participant information and demographics

Our study consisted of 44 unique subjects who participated in 1-to-6 different START NOW sessions/focus groups. The average number of START NOW sessions and subsequent focus groups was 2.09. In the end, our study had 92 different participation events amongst the 38 focus groups.

### Survey data

#### Participant demographics

36 out of the 44 unique participants (81.8%) elected to complete the demographics data, which are described in Table 3. Our participants were representative of the OBOT population in SW Virginia with a majority of Caucasian females between the age of 25–34 years old.

**Table 3:** Patient Demographics

Patient Demographics	
Characteristic	Participants ***n*** = 36, completed demographic survey (***N*** = 44)
**Distribution by age range – no. (%)**	
18–24 YO	2 (5.6)
25–34 YO	15 (41.7)
35–44 YO	7 (19.4)
45–54 YO	6 (16.7)
55–64 YO	6 (16.7)
65+ YO	0 (0)
**Female sex – no. (%)**	26 (72.2)
**Race – no. (%)**	
White	34 (94.4)
Black	1 (2.8)
Asian	0 (0)
Other	1 (2.8)
**Highest level of education – no. (%)**	
No schooling	0 (0)
Middle school	2 (5.6)
Some high school	4 (11.1)
High school	9 (25.0)
Some college	11 (30.6)
Trade/Technical/Vocational	1 (2.8)
Associate degree	6 (16.7)
Bachelor’s degree	1 (2.8)
Master’s degree	1 (2.8)
Doctorate degree	0 (0)
**Employment status – no. (%)**	
Employed	14 (38.9)
Out of work, looking for work	3 (8.3)
Out of work, not looking	2 (5.6)
Homemaker	7 (19.4)
Student	0 (0)
Military	0 (0)
Retired	0 (0)
Unable to work/Disabled	10 (27.8)

#### START NOW satisfaction questionnaire (Fig. 1)

**Fig. 2:**
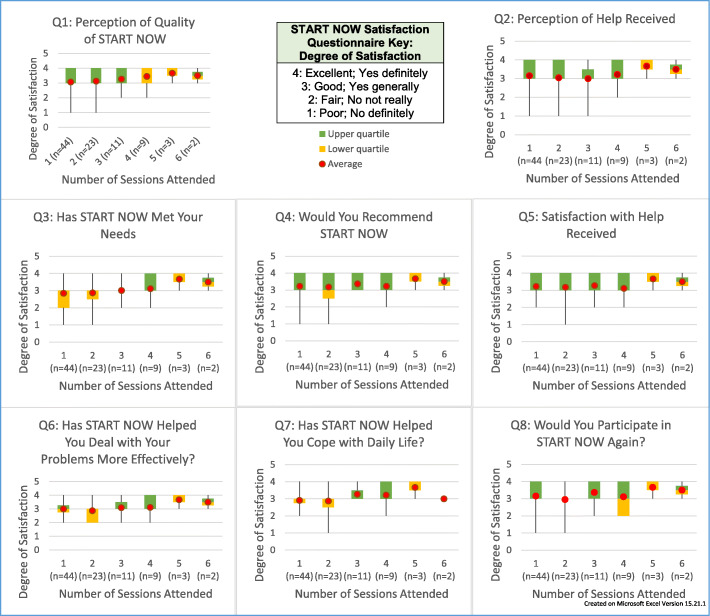
START NOW Satisfaction Questionnaire Results. With increased number of participation events (or START NOW psychotherapy sessions), there is a general increase in participant satisfaction with START NOW. This trend is also seen across all 8 questions of the START NOW Satisfaction Questionnaire

Every participant was required to complete the START NOW Satisfaction Questionnaire, an eight question survey, because the primary objective of this study is to collect patient opinion about START NOW and psychotherapy. 92 total responses were collected. Our data suggests that participants’ opinions about START NOW improve with increased participation as demonstrated in Fig. 2, which are box-and-whisker plots and indicate the maximum and minimum (whiskers), upper and lower interquartile range (green and yellow boxes, respectively), and the average (red dot).

#### START NOW assessment protocol (SNAP) (Fig. 3)

SNAP was designed to be an abbreviated, simplified behavioral marker tool, representative of the lengthier Barratt Impulsiveness Scale (BIS), Buss-Perry Aggression Questionnaire (BPAQ), and Inventory of Interpersonal Problems (IIP). SNAP was collected once for each participant during their first participation event, and the data was analyzed for percentages of responses, which is depicted in heat map format in Fig. 4. Thirty-three of the 44 unique participants (75%) elected to complete the SNAP.

**Fig. 3:**
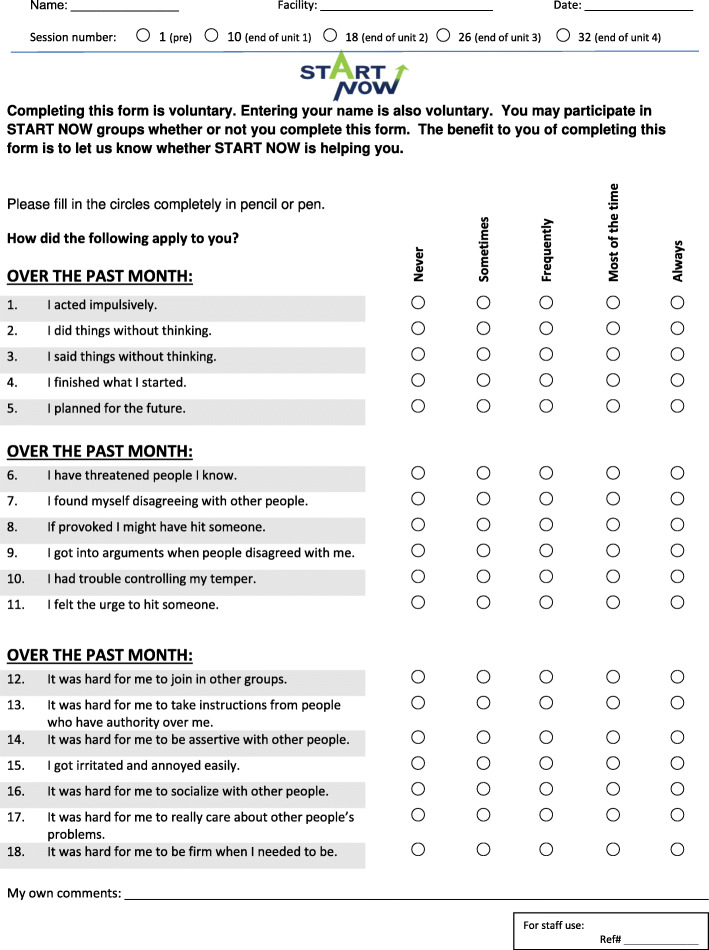
START NOW Assessment Protocol (SNAP). This is a sample image of SNAP, created as an abbreviated version of the Barratt Impulsiveness Scale, Buss & Perry Aggression Questionnaire, and Inventory of Interpersonal Problems

**Fig. 4:**
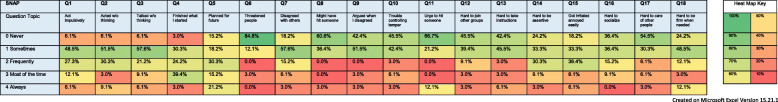
Heat-Map of SNAP Results

SNAP demonstrates several characteristics of our participants, who admit to “sometimes” engaging in impulsive behavior (Q1, Q2, Q3); however, participants were much more likely to report “frequently” failing to plan for the future (Q4, Q5). Participants also reported an aversion to physical violence (Q6, Q8, Q11). Such results may be different if SNAP was used in a population of inmates, for example.

### Audio-recorded data

As stated previously, content analysis of audio-recorded data was performed by separating questions and answers based on the five START NOW components: the real life practice exercises, the in-session practice exercises, the specific lesson of the day, the participants’ view of the clinician, and the participants’ overall impression of START NOW. Responses to questions within these five components were transcribed and rated on a five-point scale. Response rates vary per category because not every focus group participant answered questions within each component. The category “Overall Impression of START NOW” has a 100% response rate because this component is determined by a researcher upon assessing the entirety of a participant’s audio-recorded feedback. The results of this analysis is summarized in Table 4.

**Table 4:** START NOW Satisfaction According to Audio-Recorded Data

***Key****:* *5 strongly favorable,* *4 favorable,* *3 mixed,* *2 critical,* *1 extremely critical*	View of Real Life Practice Exercises	View of in- Session Practice Exercises	View of Specific Lesson of the day	View of Clinician	Overall Impression of START NOW
**Responses out of 92**	40	44	59	60	92
**Response Rate:**	43.5%	47.8%	64.1%	65.2%	100%
**3rd IQR**	5	4	5	5	5
**Median**	4	4	4	5	4
**1st IQR**	3	3	3	4	3

As described previously, researchers transcribing the audio recordings were instructed to copy every new idea (any opinion stated for the first time) verbatim. However, for every subsequent repetition of the same idea, researchers were instructed to make note of this repetition. In Table 5, the study authors include a selected sample of quotes derived from focus group audio recordings, which lend support to specific conclusions that we make for improving START NOW and its delivery. Despite the inherent bias of presenting these selected quotes in this format, we believe that readers may benefit from this qualitative data and reading audio transcriptions captured verbatim.

**Table 5:** Selected Recommendations and Supporting Evidence from Audio Recordings. This table summarizes our findings and recommendations for improving not only START NOW psychotherapy but also other psychotherapies for treating OUD

Topic	Recommendations and Conclusions	Supporting Evidence via Selected Quotes from Audio Recordings
**1. Content Strategies for New Psychotherapies**	1a: Participants want psychotherapies that target impulsivity	- “Focusing is the most important thing we’ve learned.” - “[We need help] to re-train our brain [as] addicts to learn, to think, and act more rationally.” - In regards to ABC system for functional analysis of behavior, “it’s good to bring [ABC] back up again. You need to repeat things to remember them.”
	1b: Target participants’ failure to plan for the future	- “The biggest thing that I am interested in is life things, skills in general. Skills to live life and how they can help us stay clean.” - “[Setting and Making My goals] is an important lesson of the day.” - “[It was] effective breaking down our goals, step by step, understanding how to reach it.”
	1c: Psychotherapy vignettes should be more culturally relatable and appropriate	- “I wish the material was directly correlated back to our problems.” - “There needs to be more focus on how to deal with substance abuse.”
	1d: Add more self-care skills training	- “I liked today’s session. [Self-care is important because] a lot of addicts don’t really take care of themselves. We forget.” - “I’m much less likely to take care of myself when I’m using and also taking care of myself helps me keep from using. It works both ways.”
	1e: Add more skills training for building positive relationships	- “I thought it was good, especially the “stick with the winners” [because it talked about] being around positive people and having positive people in your life to have a good structure of life.” - In regards to increasing my support system, “I really loved this session. I liked it better than any other session. It grabbed my attention.”
**2. Implementation Strategies**	2a: Improve training for all START NOW clinicians	- “There’s nothing wrong with the content. It’s all about the delivery.” - “The resident with [my doctor] seemed like she was not prepared.”
	2b: Writing down responses should be optional	- “I like to write things down.” - “I hate writing things down. I’ll never do it.” - “I can’t write because of my arm, but I really loved this session.”
	2c: Each participant should have their own personal workbook	- “It would be beneficial to take binders home and have actual things to look at home. I can think about it all I want.”
	2d: Real life practice exercises (“homework”) should be encouraged but not mandatory	- “Positive reinforcement at the end of the session will help me to motivate myself to do the practice exercises.” - “I never do the homework because I have so much going on at home.” - “Homework is better when not written. I use it at home with mom.” - “I would rather do the activity by just talking about it—not writing the homework.”
**3. Other observations**	3a: Initial resistance can be easily overcome. Subsequent session improves participants’ opinions of START NOW.	- “I thought it was more structured than other groups I have been in and the new techniques are great.” - “It was written in the workbook, but it felt real to me. I was surprised it was in the book.” - “I think that every time we have a session it gets a little better. Like when we first started this, everybody didn’t have a routine. Everybody was learning. But now it seems like it flows better.”
	3b: Clinician-patient relationships should be strong.	- “[My clinician] does a really good job. Talks to us like we are human. He tells you the truth with respect.” - “I trust [my clinician] completely.”
	3c: Treat participants as a mature audience	- “[Some content] feels a lot like what my son in fourth grade would bring home from my guidance counselor.” - “Felt childish. [The lesson] could be a little more involved and aimed towards people who are adults dealing with addiction problems.”

## Discussion

In regards to the START NOW Satisfaction Questionnaire, box-and-whisker plots suggests that participants’ opinions about START NOW improve with increased participation. But due to the small sample size and format of the study, our research lacks statistically significant proof of this trend. The heat-map analysis of SNAP demonstrates several characteristics of our participants but ultimately lacks statistical relevance as it only captures one data point.

Based on some of the qualitative data collected, we believe that our study reveals practical strategies for improving the treatment of OUD, which may be helpful to other clinicians and researchers. This data is ultimately subjective as these are select quotes from audio-transcripts. Our conclusions are summarized in Table 5, which are described again below with additional supporting evidence from other published manuscripts. We categorize our conclusions into three domains: content strategies for new psychotherapies, implementation strategies, and other observations.

With regards to content strategies, analysis of our SNAP data reveals self-reported behavioral tendencies of our participants (Fig. 4). Other researchers have identified behaviors, such as impulsivity and failure to plan for the future, that may be the most pertinent for psychotherapy to target in order to most effectively curb maladaptive behaviors contributory to substance misuse behavior [31, 32]. Comments from the focus groups also suggest that targeting impulsivity and failure to plan for the future is useful (Table 5). Audio-recorded data indicates that patients strongly want psychotherapy vignettes and examples that are culturally-relatable and appropriate (Table 5) [33, 34]. Furthermore, certain topics—self-care skills and building positive relationships—were very favorably received and thus should be emphasized (Table 5) [35].

Unlike previous qualitative evaluations, ours is unique because of START NOW’s inherent emphasis on building skills. We suggest that skill building may be the future with regards to improving psychotherapies. In START NOW, the two most practiced skills are “Focusing” and the “ABC System for Functional Analysis of Behavior,” both of which were favorably reviewed (Table 4). In general, the goal of the focusing exercises is to increase the likelihood that participants are proactive rather than reactive and impulsive. The ABC system provides a systematic method for participants to think about their actions and the consequences, to reduce and eliminate maladaptive behaviors, and to reinforce positive behaviors.

With regards to implementation strategies, patient evaluations suggest that all psychotherapy group leaders should be extremely well trained. Participants strongly preferred lessons to be delivered from experienced clinicians (as opposed to medical residents) (Table 5). Despite this, we believe that this opinion can be eliminated with residents who are well-trained in OUD, START NOW psychotherapy, and MAT OBOT [36]. We recommend intensive training with role-playing as the gold standard for preparing psychotherapy leaders. Due to participant preferences or personal disabilities, writing down responses in the workbooks should be optional (Table 5). We believe that this may enhance overall participation and engagement [33]. Participants strongly preferred having their own START NOW workbook to own, personalize, and take home. Likewise, real life practice exercises (or homework) should be encouraged but not mandatory as patients prefer increased autonomy and a nonjudgmental environment (Table 5) [37].

Other observations and conclusions from our study suggest that initial skepticism towards START NOW improves with subsequent participation with more START NOW sessions (Fig. 2, Table 5). We believe that this is an encouraging finding for other clinicians implementing new psychotherapies, which may be met with initial resistance. Overall, clinicians were viewed very favorably, even during poorly-received psychotherapy sessions, stressing the importance of strong physician-patient relationships (Tables 4 & 5). This underscores the importance of every clinician needing to build rapport and trust with their patients [37]. We believe that this is especially important despite limited time during content-rich psychotherapies such as START NOW. With regards to content delivery, participants preferred to be treated as a more mature audience with content to be delivered in a less pedantic tone (Table 5). We believe that all these recommendations may help START NOW be more accommodating and effective for treating patients with OUD.

As with other qualitative research studies, our data analysis lacks the statistical analysis and rigor expected in quantitative research. Another limitation of our study is the fact that participants completed paper surveys anonymously, and no identifiers were stated during the audio-recorded focus groups; as a result, participant characteristics and opinions cannot be correlated between the paper survey data and the audio-recorded data from the focus groups. Therefore, paper survey data and audio-recorded data is treated independently from one another. The results of SNAP are of limited utility because we only collected SNAP results at a single time point rather than at each group therapy session, for example, which would have allowed us to look at trends.

Future directions include modifying START NOW as described above and improving its delivery. Ultimately, we suggest that there needs to be a randomized controlled trial to evaluate the efficacy of START NOW psychotherapy for treating OUD. Such a study may also attempt to compare START NOW Satisfaction Questionnaire and SNAP results before and after treatment. We hope our experiences and conclusions with implementing a psychotherapy for OUD in low resource settings is useful for other clinicians and researchers trying to do the same.

## Conclusions

As described in detail in the previous section, we believe that the results of this study may guide the development and implementation of other forms of psychotherapy to ultimately improve the treatment of opioid use disorder. Our research also allows us to validate most aspects of START NOW psychotherapy modified for treating substance use disorders and revealed areas for improvement. Our study also suggests a favorable outlook of START NOW with increased participation, suggesting that the initial skepticism to this program can be overcome to allow for effective implementation. The fact that START NOW was well-received by participants is encouraging for future clinical trials exploring its effectiveness as a psychotherapy for individuals with opioid use disorder.

## Supplementary Information

**Additional file 1 Supplementary Fig. 1**. Focus Group Discussion/Question Guide.

## Data Availability

The datasets generated and/or analyzed during the current study are not publicly available due privacy concerns of releasing audio recordings and transcripts, but de-identified, modified data sets may be available from the corresponding author upon reasonable request.

## Abbreviations

ABC: Activator, Behavior, Consequence
BIS: Barratt Impulsiveness Scale
BPAQ: Buss & Perry Aggression Questionnaire
IIP: Inventory of Interpersonal Problems
MAT: Medication-assisted treatment
OBOT: Office-based opioid treatment
OUD: Opioid use disorder
SNAP: START NOW Assessment Protocol
SAMHSA: Substance Abuse and Mental Health Services Administration
