# Supplementary figures and images for: Systematic content analysis of patient evaluations of START NOW psychotherapy reveals practical strategies for improving the treatment of opioid use disorder
Source: BMC Psychiatry. 2021 Jan 10;21:23. doi: 10.1186/s12888-020-03024-x (PMC7798217; doi:10.1186/s12888-020-03024-x)

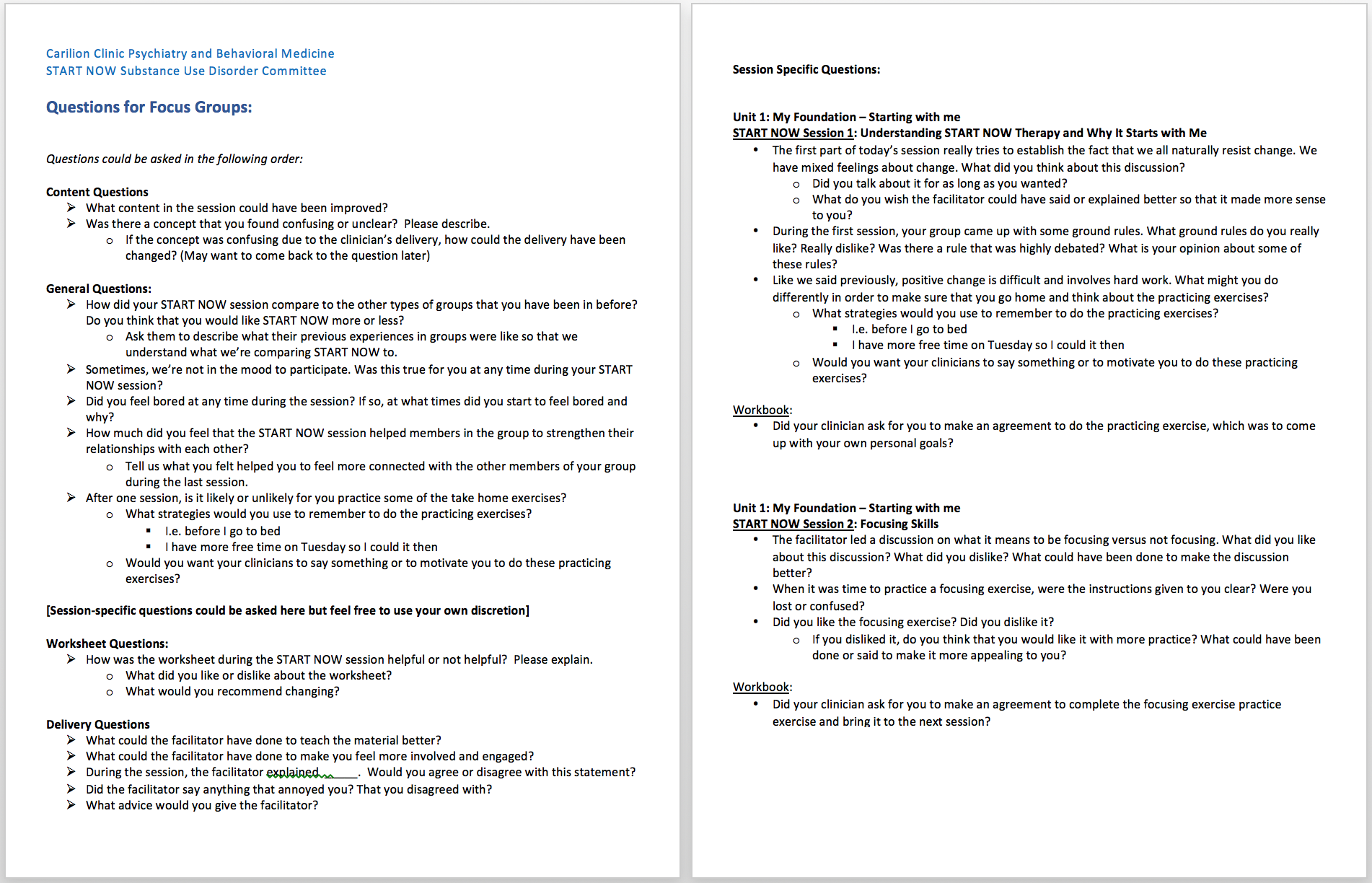

Supplement: Supplementary file 1 — Additional file 1 Supplementary Fig. 1. Focus Group Discussion/Question Guide. [file 12888_2020_3024_MOESM1_ESM.docx]
